# How Next-Generation Sequencing Is Changing Clinical Neurogenetics

**DOI:** 10.3390/neurolint13040056

**Published:** 2021-11-04

**Authors:** Daniele Orsucci

**Affiliations:** Unit of Neurology, San Luca Hospital, Via Lippi-Francesconi, 55100 Lucca, Italy; orsuccid@gmail.com

Until recently, most general neurologists were not interested in neurogenetics. When they met a patient with a suspected hereditary condition, or with a clinical picture that was presumed “too complex”, in most cases they referred these subjects to a university hospital. In these highly specialized “tertiary” centers, the patients entered a sequential diagnostic process which could last years, if the genes tested in the first instance returned negative results. In many instances, a definitive diagnosis could not be reached.

The negative implications for the patients are obvious: psychological stress, time and money consumption for reaching the referral center, and distance from the attending physician in case of need.

In recent years, next-generation sequencing (NGS) technologies have had a major impact on the diagnosis of genetic neurological diseases [1]. NGS-based panels are now widely available and provide an opportunity to analyze hundreds (or even thousands) of genes in a few weeks. These techniques, which have remarkably improved our common “diagnostic efficiency”, are also much less expensive than traditional sequencing methods (Sanger).

Therefore, in most instances, there is no need for the patient to physically go to the referral center, seeing as blood samples can be directly sent to the NGS laboratory.

Obviously, some limitations must be considered as well. The scale and complexity of these data make them difficult to interpret and require the use of sophisticated bioinformatic tools [1]. Accurate phenotypic evaluation of the patient is essential for the correct interpretation of genetic variants. In several patients, even extensive next-generation studies will not lead to a definitive diagnosis [1]. Strong communication between the clinical neurologist and the geneticist can help to address these challenges.

In this Special Issue (“The Neurogenetics of Degenerative Disorders” https://www.mdpi.com/journal/neurolint/special_issues/neurogenetics_degenerative_disorders, accessed on 1 November 2021), the genetic causes of neurological diseases, including Parkinson’s, neuropathies, motor neuron disorders, ataxias and epilepsy [2], will be reviewed. Hopefully, this issue will serve as a guide for general neurologists when they will need to approach the complex field of neurogenetics.
